# Tracking Early Systolic Motion for Assessing Acute Response to Cardiac Resynchronization Therapy in Real Time

**DOI:** 10.3389/fphys.2022.903784

**Published:** 2022-06-02

**Authors:** Manuel Villegas-Martinez, Magnus Reinsfelt Krogh, Øyvind S. Andersen, Ole Jakob Sletten, Ali Wajdan, Hans Henrik Odland, Ole Jakob Elle, Espen W. Remme

**Affiliations:** ^1^ The Intervention Centre, Oslo University Hospital, Oslo, Norway; ^2^ Institute of Clinical Medicine, University of Oslo, Oslo, Norway; ^3^ Department of Informatics, University of Oslo, Oslo, Norway; ^4^ Institute for Surgical Research, Oslo University Hospital, Oslo, Norway; ^5^ Department of Cardiology and Pediatric Cardiology, Oslo University Hospital, Oslo, Norway

**Keywords:** cardiac resynchonization therapy, left bundle branch block (LBBB), dyssynchronous wall motion, heart failure, response prediction

## Abstract

An abnormal systolic motion is frequently observed in patients with left bundle branch block (LBBB), and it has been proposed as a predictor of response to cardiac resynchronization therapy (CRT). Our goal was to investigate if this motion can be monitored with miniaturized sensors feasible for clinical use to identify response to CRT in real time. Motion sensors were attached to the septum and the left ventricular (LV) lateral wall of eighteen anesthetized dogs. Recordings were performed during baseline, after induction of LBBB, and during biventricular pacing. The abnormal contraction pattern in LBBB was quantified by the septal flash index (SFI) equal to the early systolic shortening of the LV septal-to-lateral wall diameter divided by the maximum shortening achieved during ejection. In baseline, with normal electrical activation, there was limited early-systolic shortening and SFI was low (9 ± 8%). After induction of LBBB, this shortening and the SFI significantly increased (88 ± 34%, *p* < 0.001). Subsequently, CRT reduced it approximately back to baseline values (13 ± 13%, *p* < 0.001 vs. LBBB). The study showed the feasibility of using miniaturized sensors for continuous monitoring of the abnormal systolic motion of the LV in LBBB and how such sensors can be used to assess response to pacing in real time to guide CRT implantation.

## 1 Introduction

Left bundle branch block (LBBB) causes asynchronous electrical activation of the ventricular myocardium, resulting in discoordinated contraction and inefficient pump function (Vernooy et al., 2005). Cardiac resynchronization therapy (CRT) is a widely used and effective therapy for patients with heart failure and LBBB. However, about one third of patients who receive CRT, do not benefit from the treatment and in some subgroups function may worsen after implantation (Cleland et al., 2004). Inappropriate device function may burden the patient and accrue costs to society. Ideally, identification of responders to CRT should be performed prior to implantation, and much research is focused on improving those methods. However, a method to assess acute response during the implantation of the CRT device may also have significant benefits. Acute, real-time feedback that shows how the pacing improves cardiac function will be of interest, and if no improvement is demonstrated, different locations of pacing can be tested to see if cardiac function improves. This will potentially reduce the number of non-responders due to sub-optimal lead placement. Ultimately, if the method shows no improvement of cardiac function, the implantation may be aborted. This will avoid leaving pacing wires prone to infection and clotting in the patient’s body, and as an external pacemaker is used during testing, it could save the cost of the pacemaker device and of the CRT follow-up controls that will not be needed.

However, there is yet no consensus on which hemodynamic parameter should be used to evaluate acute response (Achilli et al., 2006; Chung et al., 2008; Van Bommel et al., 2009), although promising methods exists (Odland et al., 2021). There have also been several studies testing different imaging-based criteria and although some show promising results, none have proven to add clinical value so far (Doltra et al., 2014). LV pressure measurements are a gold standard for evaluation of cardiac function and may currently be the best method for assessing response to CRT, with parameters such as maximum LV dP/dt. However, there are conflicting results regarding use of pressure as an acute response parameter (Suzuki et al., 2010; Bogaard et al., 2011). This could potentially be explained by our observation that CRT acutely reduces both end systolic and end diastolic volume (Boe et al., 2019). The reduced end diastolic volume is effectively a reduction of preload which will reduce preload dependent functional indices. An increase of an index by CRT may therefore be counteracted by a reduction by the lower preload, and the effect of CRT will be masked. There is therefore a need for a preload independent hemodynamic marker of acute response to CRT.

A commonly observed feature of LBBB is an abnormal early systolic left-right motion of the septum referred to as septal beaking or septal flash (SF) (Dillon et al., 1974). SF occurs during the isovolumic contraction period in certain heart failure patients with LBBB and is associated with reduced left ventricular (LV) pump function (Grines et al., 1989). The leftward septal motion occurs as the right ventricular free wall and septum are activated and start shortening unopposed by the late activated LV lateral wall, which in contrast passively stretches. The stretching of the lateral region increases the number of myofilament cross-bridges once activated according to the Frank-Starling effect, and hence, when it subsequently is activated, it contracts with a higher force, thus opposing the septal contraction and ultimately pushing the septum rightwards again (septal rebound stretch) (Gjesdal et al., 2011; Walmsley et al., 2015). This septal pre-ejection deformation is a complex phenomenon influenced by passive and active forces, regional contractility, electrical events and valve closure. The motion may be small or absent in the presence of septal scar, impaired global or right ventricular (RV) contractility or RV volume overload (Remme et al., 2016). However, SF assessed by echocardiography or other imaging technologies has been shown to be a reliable predictor of CRT response (De Boeck et al., 2009; Sohal et al., 2014; Risum et al., 2015; Aalen et al., 2019). The correction of this abnormal septal motion by CRT indicates an increased likelihood of LV volumetric reverse remodeling (Jansen et al., 2007; Parsai et al., 2009; Stankovic et al., 2016) and some studies have shown ability to predict long term response (Menet et al., 2017). While SF may be a clinical indicator for stratification of patients prior to CRT device implantation, there is currently no response confirmation during the intervention. This creates an uncertainty regarding response that may lead to excessive CRT implantation which burdens patients and health care systems. There is therefore a need to develop a method that improves response prediction. SF and its correction can potentially be measured during CRT implantation for acute assessment of CRT efficacy. Thus, such measurements can be used to identify in real time the patients that will benefit from the therapy and aid in the lead placement and device programming.

In this proof-of-concept study we investigated if the SF motion could be used as a measure of acute response to CRT and propose a method for real-time measurement of the motion that can be shown on a monitor during implantation. The first hypothesis of the study was that SF would be reduced or totally abolished with optimal CRT. A second hypothesis was that this method could also identify optimal LV lead placement. Finally, the third hypothesis was that these measurements could be performed using miniaturized electromagnetic (EM) tracking sensors. EM sensors are commonly used in humans for tracking catheter positions in the body (Nafis and Jensen, 2008; Boutaleb et al., 2015; Beaulieu et al., 2017), and these coil sensors are very small and can be potentially incorporated in the CRT pacing leads or guiding wires. This combination of lead and sensor has already been proven possible with devices such as SonRtip^TM^ which consists on an accelerometer embedded in the atrial lead. The sensor then measures mechanical vibrations to optimize the CRT timings (Brugada et al., 2017). Thus, EM tracking sensors could be integrated in the pacing leads in a similar way, giving a continuous measurement of displacement. Alternatively, temporary insertion of EM sensors on the right side of the septum and in a coronary vein on the LV lateral wall during implantation by incorporating EM sensors in the guide-wires or using EM-catheters, could be used to track the septum and LV lateral wall positions for measurements of SF during implantation. There are already other invasive methods that similarly use catheters to study CRT response by electro-mechanical mapping of the heart and studying the electrical activation pattern in the ventricle (Gyöngyösi and Dib, 2011; Grace et al., 2019). The study was done in a canine model with LBBB comparing responses to different pacing configurations and lead placement during CRT. Implanted sonomicrometry crystals were used as gold standard to measure SF and test the first two hypotheses. As a proof of concept, we also attached EM sensors in the septum and on the LV lateral wall to mimic a clinical setup and test the third hypothesis if this sensor system could be used for acute assessment of SF, comparing it to the gold standard sonomicrometric measurements.

## 2 Materials and Methods

### 2.1 Animal Preparation

**FIGURE 1:**
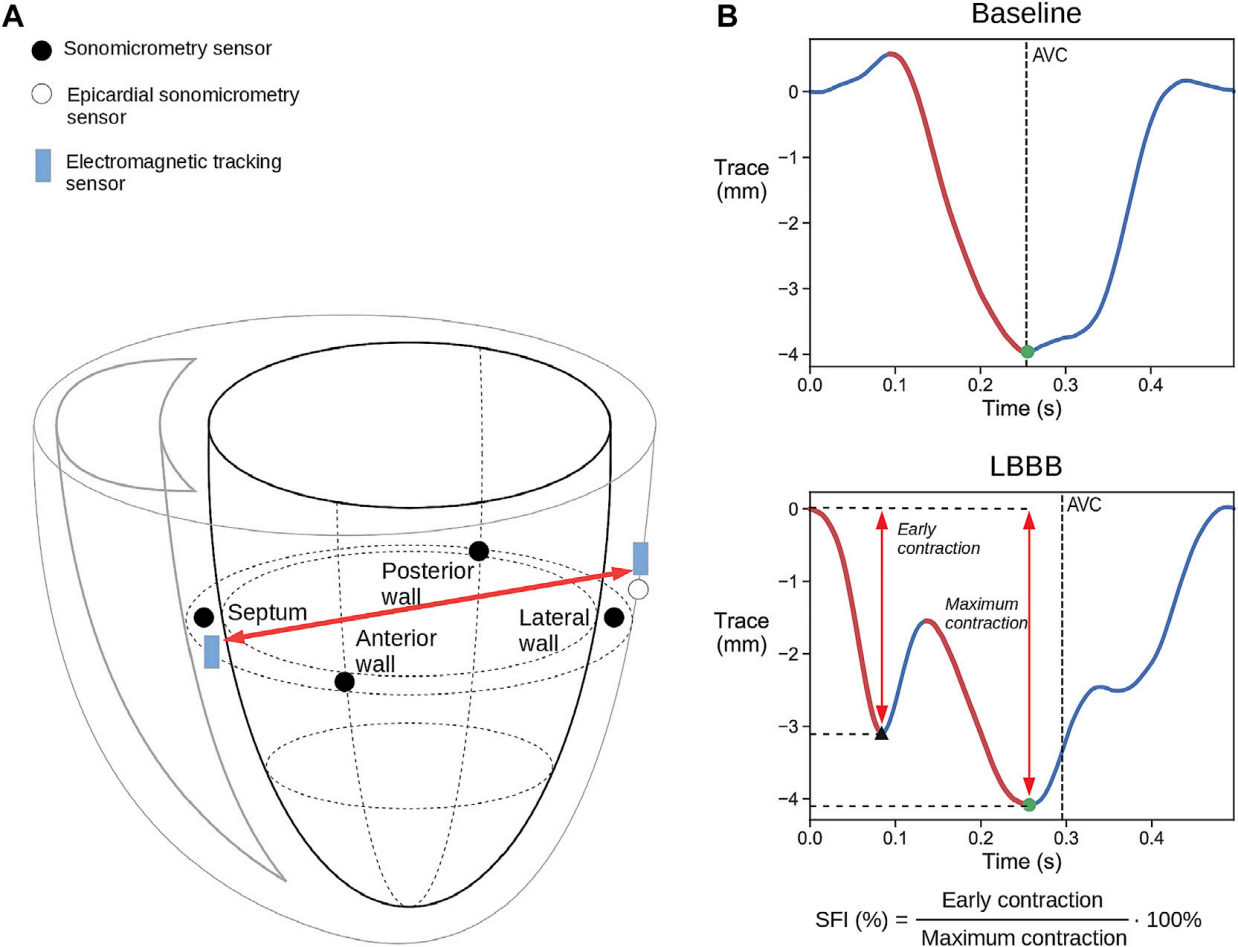
**(A)** Schematic illustration of placement of the combined sonomicrometry crystals and IM-EMG sensors and the electromagnetic tracking (EM) sensors. The red arrow indicates the change in the distance that was measured between the sensors. Only one of the three epicardial LV free wall EM and sonomicrometry sensor pairs are shown for simplicity. **(B)** Representative displacement traces measured with EM tracking sensors during baseline and LBBB. The red traces mark the contraction during systole. The black triangle marks the end of the early contraction, while the black dots mark the point where full contraction is achieved which is then used to calculate SFI. AVC = aortic valve closure.

Our group has performed several studies on LBBB and CRT where sonomicrometric crystals have been implanted which allows analysis of SF (Gjesdal et al., 2011; Aalen et al., 2019; Andersen et al., 2021). This study was therefore a combination of retrospective analysis of previously performed experiments (*n* = 4) and a prospective study where EM tracking sensors were implanted (*n* = 22). Thus, a total of 26 mongrel canines (8 female) of average weight 32 kg (±3 kg SD) were used in acute experiments for validation of the measurement of motion during pre-ejection period with sonomicrometry and EM tracking sensors. The study was conducted according to the guidelines of the Declaration of Helsinki, and approved by the Institutional Review Board (or Ethics Committee) of The Norwegian Food Safety Authority (FOTS ID: 8628, date of approval: 03.10.2018). The animals were supplied by the Center for Comparative Medicine (Oslo University Hospital, Rikshospitalet, Oslo, Norway). The animals were ventilated, anesthetized by propofol/opioids and surgically prepared as previously described (Andersen et al., 2021), including partial splitting of the pericardium from apex to base and loose re-suturing of the pericardial edges after completed instrumentation. LV pressure was measured with a calibrated micromanometer-tipped catheter (MPC-500, Millar Instruments Inc., Houston, TX) which was drift adjusted using a fluid-filled catheter in the left atrium (Andersen et al., 2021). LV volume was measured by sonometric crystals (Sonometrics, London, Ontario, Canada). Crystals were implanted subendocardially in a long axis diameter pair (apex to base), and two short axis diameter pairs in the LV equator (posterior to anterior wall and septum to lateral wall) (Figure 1). From these three diameter pairs the continuous volume was estimated using the formula (Mercier et al., 1982):

$$V=\pi /6\cdot \left(longaxisdiameter\cdot shortaxisdiameter_{1}\cdot shortaxisdiameter_{2}\right)$$

Stroke work was then calculated as the area of the LV pressure–volume loop. The four crystals placed in the equatorial plane were equipped with electrodes for measuring intramyocardial electromyograms (IM-EMG) to assess regional electrical activation times of the LV.

An epicardial pacemaker lead was attached to the right atrium, allowing measurements at a fixed heart rate. RV and LV pacing leads were placed to facilitate CRT by biventricular pacing. The endocardial RV lead was placed on the septum in the RV apex, while three epicardial pacing leads were placed on the LV free wall: in a lateral position, in an apical position and close to the base on the anterior wall. The reason for placing three LV leads was to allow biventricular pacing from different LV locations to vary the degree of improvement. One EM tracking sensor (3DGuidance trakSTAR 2, NDI, Waterloo, ON) was inserted into the septum near the septal sonomicrometric crystal. Additionally, another pair of sonomicrometric and EM tracking sensors where sutured to the LV lateral wall. This allowed measurements of the diameter between the septum and the LV free wall with both sensors (Figure 1). A Mid-Range Transmitter used as reference for the EM tracking system was placed next to the animal and its x-axis was aligned with the longitudinal axis of the heart.

### 2.2 Experimental Protocol

Data were obtained at a fixed heart rate by atrial pacing (AP) at 120 beats per minute in all settings to avoid alterations in hemodynamic response parameters from differences in heart rate alone. After baseline recordings, LBBB was induced by radio-frequency ablation (Celsius Catheter, Biosense Webster, Inc.), with confirmation of successful induction by QRS widening, limb lead R wave notching and LV contraction patterns. When applying CRT, different pacing locations were tested. As CRT decreases both end-systolic and end-diastolic volumes, effectively reducing preload (Boe et al., 2019), it may mask the improvement by CRT. To correct for the acute changes in preload, we calculated the hemodynamic indices at identical end diastolic volume (EDV) for the different settings in each animal. Heart beats with identical EDV were found from transient vena cava constrictions that were performed in all settings. The preload corrected stroke work, SW_EDV_, was obtained from the beats with the highest common EDV values from baseline, LBBB and CRT recordings. SW_EDV_ was then used as an index of global cardiac performance. All pressures, sonomicrometry, and EM tracking data were recorded simultaneously; EM data at 250 S/s and the other data at 200 S/s.

### 2.3 Signal Processing and Analysis

We used the Python programming language [version 3.7, Python Software Foundation (Van Rossum, 1995)] for all signal processing. All recordings were done with the respirator switched off to ensure that the values were unaffected by changes due to respiration. The raw EM signals were filtered using a second order Savitzky-Golay filter with a window size of 51 samples, to smooth them and remove high frequency noise.

### 2.4 Cardiac Function Estimation

The SF index (SFI) which was used to examine if CRT was able to correct the dyssynchrony, was calculated as the early systolic shortening of the LV septal-to-lateral wall diameter divided by the maximum shortening achieved after the early systolic motion during the cycle (Figure 1B). This diameter was measured by sonomicrometry from the septal crystal to the epicardial crystal next to the LV lateral wall pacing electrode, as shown in Figure 1A. Similarly, the spatial coordinates of the EM sensor in the septum and the EM sensor next to the LV lateral wall pacing electrode were used to calculate the equivalent diameter between the EM sensors.

### 2.5 Electromagnetic Sensor Validation

To check the accuracy of the EM tracking sensors and study its ability to measure the SFI, we compared the diameter trace and the derived SFI with the ones obtained with sonomicrometry.

### 2.6 Statistical Analyses

All statistical analyses were computed with SPSS software (version 28; SPSS Inc., Chicago, Ill). No statistical power calculation was conducted prior to the study as it was intended as a proof of concept. The sample size in this study is therefore relatively low and the statistical tests must therefore be considered with caution. Normality of distributions was determined using Shapiro-Wilks test. To test for significant effects of the interventions we used two-tailed Student’s paired sample *t*-test on those with normal distribution and Wilcoxon signed-ranks test for the rest. Statistical significance was determined as *p* < 0.05. All values represent the mean of five consecutive heart cycles except data collected during transient caval constriction where only one beat was used. Values are reported as mean ± SD. No outliers have been excluded from the statistical tests.

A total of 26 experiments were conducted. Out of all of them, a total of 8 were excluded due to failure to induce LBBB or due to equipment malfunction. The protocol of each experiment varied slightly, so that only 14 out of the remaining 18 experiments included caval occlusions that allowed for preload adjustment of volumetric measurements, i.e. SW_EDV_. Only 12 of these experiments had EM tracking sensors connected.

## 3 Results

### 3.1 Cardiac Function Estimation

Hemodynamic values from baseline, LBBB, and the three CRT positions of all 18 experiments are shown in Table 1. Notably, there was a significant, acute reduction in EDV when CRT was turned on, regardless of the position of the lateral lead. Maximum LV dP/dt was increased for all three CRT positions. There was no significant change in stroke work, while stroke volume, cardiac output and ejection fraction were only significantly improved for CRT with apical position. On the other hand, when acute changes in preload were corrected for by measurements at similar EDV, there was a significant improvement with all CRT positions for all indices including stroke work (SW_EDV_).

**TABLE 1:** Hemodynamic values at baseline, LBBB, and all biventricular pacing sites (CRT) for all animals.

	Baseline	LBBB	CRT—lateral wall	CRT—apex	CRT—base	*n*
LV end-diastolic volume (ml)	71 ± 24	76 ± 26‡	72 ± 25*	73 ± 28§	72 ± 29§	18
Sensor indices						
Septal flash index (%)	9 ± 8	88 ± 34†	21 ± 20*	13 ± 13*	24 ± 22‡*	18
Septal flash index from EM tracking sensors (%)	9 ± 11	68 ± 49‡	9 ± 15§	6 ± 10§	18 ± 23§	12
Hemodynamic functional indices						
Stroke work (mmHg·ml)	1,020 ± 317	911 ± 283	796 ± 332‡	963 ± 331	873 ± 335‡	18
Stroke volume (ml)	13 ± 4	11 ± 3	11 ± 4‡	13 ± 3§	12 ± 4	18
Cardiac output (ml/min)	1,534 ± 408	1,414 ± 388	1,302 ± 448‡	1,581 ± 457§	1,428 ± 548	18
Ejection fraction (%)	19 ± 4	16 ± 5‡	16 ± 6‡	19 ± 5§	17 ± 4‡	18
LV dP/dt_max_ (mmHg/s)	1,568 ± 363	1,374 ± 276‡	1744 ± 744§	1,694 ± 705§	1711 ± 614§	18
Preload corrected hemodynamic functional indices						
Stroke work (mmHg·ml)	917 ± 279	541 ± 204†	748 ± 345‡§	848 ± 331*	792 ± 324§	14
Stroke volume (ml)	11 ± 3	7 ± 2†	9 ± 4§	11 ± 3*	10 ± 3§	14
Cardiac output (ml/min)	1,327 ± 316	810 ± 262†	1,073 ± 456‡§	1,300 ± 408*	1,195 ± 388§	14
Ejection fraction (%)	17 ± 3	10 ± 3†	14 ± 6‡§	17 ± 4*	16 ± 5§	14
LV dP/dt_max_ (mmHg/s)	1,639 ± 383	1,319 ± 234‡	1871 ± 712§	1758 ± 566§	1854 ± 496§	14

Values are mean ± SD. †*p* < 0.001 compared to baseline, ‡*p* < 0.05 compared to baseline, *p < 0.001 compared to LBBB, §*p* < 0.05 compared to LBBB., Abbreviations; LBBB, left bundle branch block; CRT, cardiac resynchronization therapy; LV, left ventricle; LV dP/dtmax–maximum time derivative of left ventricular pressure; EM, electromagnetic.

**FIGURE 2:**
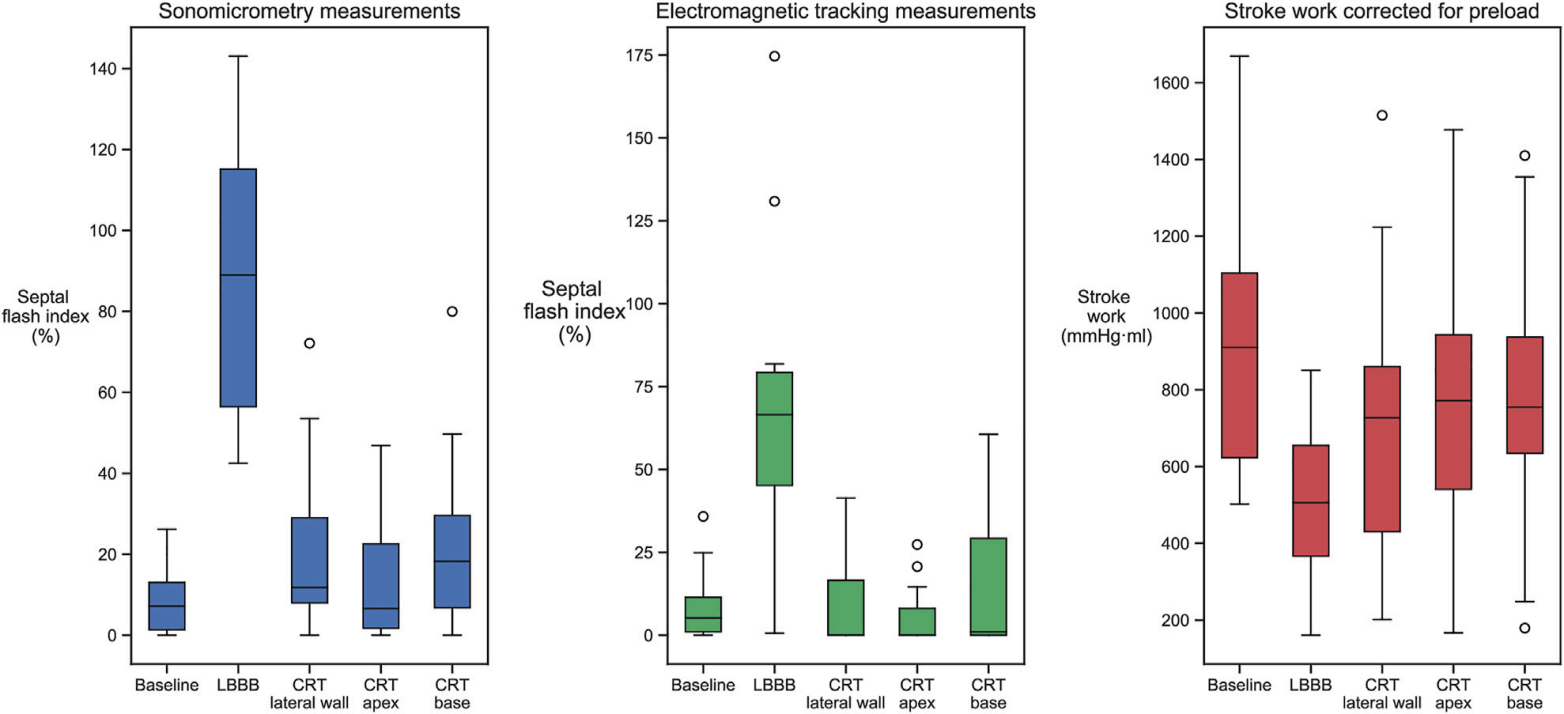
Septal flash index, measured by sonomicrometry and EM sensors, and SW_EDV_ values measured in baseline, LBBB, and all three biventricular pacing sites. Induction of LBBB changed all measurements from baseline, while CRT returned them closer to baseline values.

At baseline, with normal electrical activation, there was limited early-systolic shortening, and SFI by sonomicrometry was low (Table 1). After induction of LBBB, this shortening and the SFI significantly increased. Subsequently, CRT reduced SFI close to baseline values. There were no statistical significant difference in degree of improvement between the three CRT positions. However, as seen in Table 1, the trend was that apical position generated the highest preload corrected stroke work (*p* = 0.096 vs. lateral position) which was reflected also by a trend of lowest SFI value at this position (*p* = 0.06 vs. lateral position). The SFI measured by the EM sensors showed qualitatively a similar pattern as measured using sonomicrometry and furthermore, reflected the corresponding changes in cardiac function by SW_EDV_ (Table 1; Figure 2).

### 3.2 Electromagnetic Sensor Validation

**FIGURE 3:**
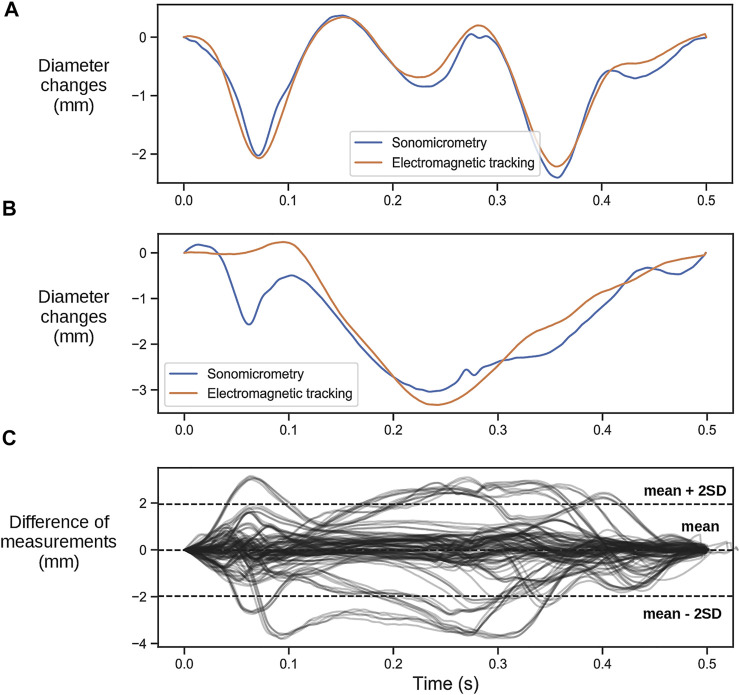
**(A)** Representative case of a heartbeat comparing the change in the left ventricular septal-to-lateral wall diameter measured with the two different sensors where the measurements of the EM tracking sensors align with the ones obtained with sonomicrometry. **(B)** Representative case where the EM tracking sensors did not capture the rapid early contraction. **(C)** Difference between diameter changes measured with sonomicrometry and EM sensors for all recordings.

The correspondence between the LV septum-to-lateral wall diameter trace measured using sonomicrometry and the one measured using EM sensors varied substantially between cases. In some cases, there were excellent correspondence (Figure 3A), where the SFI measured by the two methods were practically identical. However, there were cases with varying discrepancies where the EM measurements did not capture the rapid motions of the SF very well (Figure 3B). We noticed that the EM sensors were not properly sutured to the heart in some cases, and therefore was displaced or did not follow the motion of the heart correctly. The EM sensors we used were not designed for this purpose as they are intended to be embedded into medical instruments such as catheters, endoscopes, guide wires, and needle tips in order to help localize the instrument while navigating through anatomical tracts. For this purpose, they are made with a thin and stiff wire. Two sutures approximately 1 cm apart were used to attach the wire to the epicardium. However, the stiffness of the wire and lack of proper attachment points resulted in dislocation of the wire during some experiments. This resulted in improper contact of the sensor to the point it was initially sutured to the heart and hence improper tracking of the motion. The difference between the measured diameter trace by sonomicrometry and the EM sensor for all the 12 animals with EM sensors are shown in Figure 3C.

## 4 Discussion

In this study we have shown that acute changes in LV function during CRT implantation can be measured with sensors attached to the myocardium by assessing the degree of abnormal systolic motion. A hallmark of LBBB is the large pre-ejection shortening in the early activated septum and the resulting shortening of the LV septum-to-lateral wall diameter. Despite mechanical measurements not being currently recognized as relevant for the selection of patients for CRT, correction of electrical dyssynchrony should result in improved mechanical function for a meaningful response to occur. Successful CRT will synchronize the LV and remove or reduce this abnormal pre-ejection shortening. Additionally, patients with a greater septal to lateral wall delay present a more evident mechanical dyssynchrony (Andersson et al., 2013), which supports the notion that a mechanical index could be used as marker of dyssynchrony and monitor the effect of CRT. This is also supported by the notion that presence of SF is a statistically significant independent predictor of CRT response and that its correction by CRT is associated with significant acute and chronic benefits (Gabrielli et al., 2014; Walmsley et al., 2015). We therefore believe a mechanical measurement should be of potential use. In addition to this, SF is a relatively well defined and distinctive contraction pattern that is easily recognizable, so we believe that presence of SF and its abolishment or reduction would be a good marker of response to CRT. Hence, we studied if two sensors placed on the septum and on the LV free wall could measure this abnormal motion and if it would be able to monitor the response to CRT. We assessed this by calculating SFI as the proportion of the early systolic shortening relative to maximum shortening during ejection. The results of our study showed a reduction of this index with an improvement in LV function, which agrees with clinical data showing that more synchronous contraction during the pre-ejection period is associated with a better long-term clinical outcome (Odland et al., 2021). Note that as we extracted this index from the entire diameter, it is not strictly a pure septal motion as the originally proposed SF.

**FIGURE 4:**
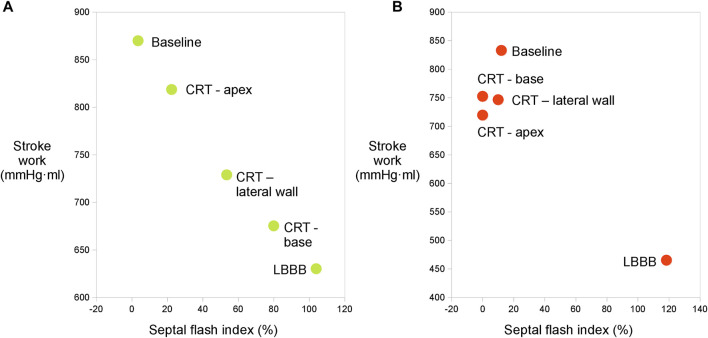
**(A)** Relation between SW_EDV_ and SFI calculated with sonomicrometry from one experiment where different ranges of response were measured from different LV lead placements. There is a clear trend towards a reduction of SFI with improvement of LV function. **(B)** Relation between SW_EDV_ and SFI calculated with sonomicrometry from one experiment where the points obtained through different lead placements are clustered together.

While the measurements showed a distinct and significant improvement in function from LBBB to CRT, we were not able to produce significant difference in the degree of improvement between the three LV lead positions. There was a smaller difference in most of the hemodynamic values, such as maximum dP/dt than we initially expected. We can only speculate as to causes of this, which could be due to differences in heart size or in the electrical conduction system between canines and humans, or short distance between the alternative lead positions. Humans who are treated with CRT, are usually in heart failure with enlargement of the heart size and may also have impaired conduction within the left ventricle, hence, different lead positioning is expected to broadly impact resynchronization. The lack of difference in response prevented us from investigating the ability of the method to identify optimal lead placement or the correlation between SW_EDV_ and SFI. This is showcased in Figure 4 where we show the data from an experiment where different pacing positions gave different ranges of response (Figure 4A) and another in which similar values were obtained for all lead placements (Figure 4B).

As this was a proof-of-concept study, the intention was not to report the actual diagnostic accuracy, but rather show the potential of this method. Sensors for this purpose need to be custom made for it; small and robust enough to be added to the equipment that is routinely used in CRT implantation without adding complexity to the implantation procedure. For this purpose we chose to study EM tracking sensors, which can be miniaturized and are already used in different clinical setups where they can be visualized as they are navigated through different anatomical tracts in real time. However, the commercially available sensors we used, were not designed for our purpose, and the challenges when attaching them and recording data affected the accuracy and reliability of the measurements in some experiments. As a result, while the distance measured between the EM tracking sensors showed agreement with the reference in most cases, there were cases where the two technologies showed differences. Despite this, as we are aware of the limitations of the technology we used, we argue that by solving these issues with a custom design, the measurements would agree fully with those of sonomicrometry and the method would therefore work as well. Importantly, the EM sensor needs a higher sampling rate for this purpose than what may be typical for other clinical use of EM sensors. In two pilot experiments, we used a different EM tracking system, Aurora™ (NDI, Waterloo, Ontario, Canada). Those sensors had a sampling rate of 40 S/s, which did not allow an accurate tracking of the rapid motions during septal pre-ejection deformation. Data from those experiments could therefore not be used for the study, and the EM tracking system was changed to the one described in section 2.1.

### 4.1 Clinical Implications

The proposed SF index in this study, is able to act as an indicator for acute changes in LV function during CRT. This index could become a simple and reproducible tool for clinicians to assess baseline contraction characteristics and acute effects from CRT. The change in SFI could furthermore inform clinician about optimization strategies when testing capture and response to pacing. By measuring the reduction of SFI in real time, a clinician can determine whether the therapy is having the desired effect. If there is no reduction, or if it is a minor one, other pacing settings or lead positions should be tested. Ultimately, the baseline SFI characteristic or the SFI response to pacing could provide necessary information for the operator to avoid implantations associated with poor clinical outcomes (Ross et al., 2020). If the EM tracking sensors are permanently placed with the pacemaker by incorporating them in the pacing leads, the system could assist in follow-up assessment to potentially optimize the programming of the pacemaker. This will also have the benefit of requiring no additional invasive procedure to insert them into the patients. However, in this case the pacemaker will have to be more complex to incorporate the extra wiring. Alternatively, the sensors may be introduced independently during CRT implantation only. This will then represent an extra invasive burden for the patient, though potentially this burden may be reduced if the sensors are incorporated in guidewires that will anyhow be introduced in the patients during the procedure.

Nowadays, SF can be reliably assessed by echocardiography, which is a non-invasive and harmless alternative. However, it seems less practical for use during CRT implantation as it requires extra personnel, time to obtain images and time for post-processing the images to quantify the desired indices as well as extra space in the operating-room for the ultrasound equipment. Another limitation when measuring SF by echocardiography, is the inter- and intra-observer variability. By using standardized mechanical devices, such as EM tracking sensors, this variability could be largely omitted. Hence, an automated sensor system for real-time analysis of the SF pattern seems a more attractive alternative which may become an important tool during CRT implantation.

### 4.2 Limitations

The present study used data from long interventions performed on heavily instrumented animals under anesthesia, hence heart function was depressed also at baseline. Furthermore, sonometric crystals were used to calculate LV volume. These crystals were not placed on the endocardium but somewhere in the wall and hence the LV cavity volume calculations were exaggerated as it included some myocardial mass and as a result the derived ejection fraction values were underestimated. We were not able to obtain statistically significant different responses from different pacing sites in our animal model. Hence, we were only able to evaluate correct capture and response to CRT, while the method’s ability to guide optimal lead placement needs to be further investigated. As previously discussed, the EM sensors used in this study presented some limitations and did not always reflect accurately the heart wall motion. Hence, different sensors should be tested in future studies to find more suitable ones for this purpose.

## 5 Conclusion

This study showed first that measurements of the septal flash index in the LV septal-to-lateral wall diameter can be used to evaluate the acute improvements in LV function by CRT, and secondly that electromagnetic tracking sensors can be used for measuring this index. Such technology could thus have a role for assessing acute response to CRT and guide implantation.

## Data Availability

The raw data supporting the conclusion of this article will be made available by the authors, without undue reservation.
